# Monthly Summary

**Published:** 1883-06

**Authors:** 


### MONTHLY SUMMARY.

THE CARE OF THE TEETH.—The teeth not only materially aid the processes of digestion and thereby promote health and comfort, but when properly cared for contribute largely to the good appearance of the mouth and to the expression of the face. Nothing is more ornamental and attractive in the features of an individual, male or female, than white, evenly-arranged and shapely teeth. Apart from appearance's sake, in respect to which most people are always jealous, the comfort of a clean mouth and the consciousness of a pure breath are especially to be desired by every one.

We would naturally suppose that everyone would desire to possess clean and healthy teeth did we not know on the contrary that very few persons really prize these truly important organs. The teeth are sadly abused by many. The tooth-brush is a poorly appreciated article of domestic comfort, often not used at all, or if used the application is frequently ineffective and irregular. As for the dentist he is too often shunned and dreaded as an evil genius until relentless pain forces an acceptance of his cruel steel. The fact is very few people know how to take care of their teeth or appreciate their importance to the economy. They have failed to learn the fact that "prevention is better than 'cure' and in consequence neglect the teeth until it is frequently too late to save them from total decay.

It may be that the cost of preventive treatment is an item considered by many; nevertheless this item of expense should be viewed from the standpoint of the old maxim "a stitch in time saves nine." It is poor economy to delay the care of an important organ until that organ is too badly damaged to be of much further service. The proper time to learn to take care of the teeth is in youth. Children should early be taught to use the brush and to guard against the use of agents which injure or discolor the teeth. A good brush should be selected and the child should be instructed to use it properly and regularly. A brush to be of service should be of suitable size for the mouth, with elastic bristles having serrated ends. The brush should be pressed against the teeth and then brought backwards and forwards with such gentle motion as will force out any accumulations which have lodged between them. Too great force with a brush only scratches the enamel and leaves the debris between the teeth undisturbed. The proper time for brushing the teeth is after meals, but as it is not always convenient to use the brush after each meal it is better to discharge this duty just before retiring at night. The teeth may be brushed in the morning to remove the sordes which have accumulated during the night, as also to cleanse the mouth and purify the breath. As a means of guarding against the consequences of neglect, every one should make a semi-annual or annual visit to a skilled and honorable dentist to have the teeth carefully examined and treated, if required.

In view of the great importance of good teeth to everyone, the physician should guard his patients against the neglect of these organs and instruct them in their hygienic management. the matter is of sufficient importance, in our opinion, to deserve this lengtlhy notice.—*Md. Med. Journal.*

---

"UNDER THE SURGEONS TOUCH."—Dr. Moses Gunn, Prof. of Surgery in Rush Medical College, Chicago, while conducting a surgical clinic on March 10, 1883, introduced to the class an infant, 10 weeks old, named Willie Haas, who was suffering from a nævus, situated near angle of mouth and about the size of a fifty cent piece. The child, according to Prof. Gunn's statement, was imperfectly developed and the growth of the vascular tumor was rapid. The injection of a solution of chloride of iron, a remedy used by the Professor in many other cases, was selected as a means of relief. Immediately after the injection of 3 drops of the solution, the child became slightly convulsed and died within thirty minutes. Prof. Gunn's explanation of this unfortunate termination was that the babe died of shock. An inquest was held upon the remains and we are glad to record the fact that Prof. Gunn was exhonorated from blame. The old adage "He jests at scars that never felt a wound" is often exemplified in the practice of surgery. But all who are acquainted with the skill attainments and superior abilities of this distinguished surgeon will at once realize that no foresight, however, keen, no precaution, however, complete, could have prevented this sad termination. That death occasionally takes place in spite of every care and attention is an unanswerable argument in favor of the use of safer agencies when they are available. When several remedical agents possessing equal curative powers, are at the command of the surgeon, he is not always at liberty to select the one whose use is attended with the least danger, for the reason that many other matters present themselves for consideration in determining the selection. Hence in the practice of busy surgeons, instances occur almost daily in which these dangerous measures are not only indicated but demanded. The report of this case while it reflects no discredit upon the eminent gentleman in whose hands it occurred, will we hope serve to encourage other surgeons to weigh carefully evidence in favor of and against all dangerous remedies and procedures before putting them into use.— *Western Medical Reporter.*

---

A RARE CASE OF EXOSTOSIS.—Dr. L. M. Mathews, of Fort Scott, Kansas, writes to the *Ohio State Journal* as follows.

On the 21st of March, 1883, Mrs. B., age 56, called at my office for the purpose of having the remaining eight superior and one inferior tooth extracted, preparatory to having artificial sets inserted, they being badly decayed. The first tooth I extracted was the right superior, lateral incisor, which required a great deal of force to remove it. I found a square shoulder formed, about half way from the neck to the apex, the enlargement extending to the apex, making the exostosed portion fully one half larger in diameter, than the part above it. I then removed the first bicuspid, on the same side. I found the same condition of osseous formation. I then removed the canines, and found them in the same condition. I then removed the first and second molars, on either side. The roots were nearly obliterated by the osseous deposit. The lady informed me that all her other teeth had been in the same condition. I have, at different times, in a practice of ten years, removed as many as two teeth in the same mouth that were exostosed. I do not remember reading of a case where the entire denture was exostosed. This may not be a rare case, but I thought it might be well to present it, if not for the information of my confreres, for their opinions. The lady said she had never suffered very much with her teeth, but had been a great sufferer for several years, by neuralgic pains in her eyes and forehead, with quite a weakening of the optic nerve. These pains were not periodical, as is usual, but constant.

Is this neuralgia, and the partial loss of sight, a sequence of the exostosed condition of the teeth? I shall watch the case with interest, and expect an improvement in the eyesight, and the disappearance of the pains in the forehead.

---

FOREIGN BODIES IN THE AIR PASSAGES.—Dr. L. R. Weist says in a paper read at the late meeting of the American Medical Association, tabulates his conclusion as follows:

1. When a foreign body is lodged either in the larynx, trachea or bronchi, the use emetics, errhines, or similar means should not be employed, as they increase the sufferings of the patient, and do not increase his chances of recovery.

2. Inversion of the body and succession, though sometimes useful, are dangerous, and should not be practiced unless the windpipe has been previously opened.

3. The presence simply of a foreign body in the larynx, trachea or bronchi does not make bronchotomy necessary.

4. While a foreign body causes no dangerous symptoms, bronchotomy should not be performed.

5. While a foreign body remains fixed in the trachea or bronchi, as a general rule, bronchotomy should not be practiced.

6. When symptoms of suffocation are present, or occur at frequent intervals, bronchotomy should not be resorted to without delay.

7. When the foreign body is lodged in the larynx, there being no paroxysms of strangulation, but an increasing difficulty of respiration from edema or inflammation, but bronchotomy is demanded.

8. When the body is movable in the trachea, and excites frequent attacks of strangulation, bronchotomy should be performed.

---

HARDENING PLASTER CASTS.—When it is desirable to preserve a plaster cast, or when it is important that it should not be marred and injured by handling, it may be hardened by boiling it for half an hour in a strong solution of potash alum. If such a preparation be kept at hand, ready for use when required, it is but little trouble or labor to make models that are almost like metallic casts, and much of the annoyance and vexation of laboratory work may thereby be avoided.

Another method of hardening is to thoroughly dry the plaster, and paint it over repeatedly with thin shellac varnish, drying the cast between the application of the different coats, until saturation is accomplished.—*Independent Practitioner.*

---

OUR DENTAL BRETHERN on both sides of the water are becoming a very pushing class. Not long ago several Parisan dentists offered to do dental work free in the municipal hospitals. The Municipal Council, politely refusing this over-liberal offer, reminded the petitioners that it was a principle of true democracy that every man should be paid for his work. New York dentists vent their surplus activities in other ways. A firm in this city, for example, have a large show-window containing a wax figure with moving jaws and a hysterical smile. Above it is the legend : " Archer, Puller, & Filer, French Dentists. No charge for extracting teeth. Come in and have one drawn. Rubber sets, $1 to $5."—*Med. Record.*

---

A SIMPLE METHOD OF PRODUCING LOCAL ANÆSTHESIA.—Dr. Cheize relates (*Moniteur de la Policlinique*, March 25, 1883) a simple procedure adopted by him in a case of ingrowing toe-nail, requiring immediate operation, at a time when he had no apparatus at hand. He saturated a little piece of lint with ether and placed it on the toe. He then projected the air from an ordinary pair of hand-bellews upon the lint until the ether was evaporated, This was repeated two or three times, when anæsthesia was so complete that the nail was removed without the patient's knowledge.

---

DENTAL FORMATION IN THE NASAL CAVITY.—Dr Max. Schaffer relates the following case in the *Deutsche Medical Wochenschrift*, No. 2, 1883: " A gentleman had for some time experienced a slight obstruction in one nostril. Examination revealed the presence of a hard, roundish, movable body, which was attached to the floor of the nostril about one inch from the anterior opening. It was removed by the snare, and was found to be a perfectly formed canine tooth, a little over an inch in length. The anterior portion was covered with enamel, and its little root presented a layer of cartilage. There was no bony alveola. The patient had all his teeth."

